# Puncture approaches and guidance techniques of radiofrequency thermocoagulation through foramen Ovale for primary trigeminal neuralgia: Systematic review and meta-analysis

**DOI:** 10.3389/fsurg.2022.1024619

**Published:** 2023-01-06

**Authors:** Huabo Liu, Lulu Xu, Wensheng Zhao

**Affiliations:** ^1^ Department of Pain Medicine, Zhoushan Hospital of Zhejiang Province, Zhoushan, China; ^2^ Department of Pain Medicine, Hang Zhou Red Cross Hospital, Hangzhou, China

**Keywords:** trigeminal neuralgia, radiofrequency thermocoagulation, foramen ovale, puncture approach, guidance technique

## Abstract

**Objective:**

Trigeminal neuralgia (TN) is one of the leading causes of facial pain and seriously affects patients' quality of life. Foramen ovale (FO) radiofrequency thermocoagulation is a classic approach for the treatment of TN that has failed pharmacological therapy. This study summarized the safety and efficacy of transforaminal radiofrequency thermocoagulation for TN by comparing puncture approaches or guidance techniques, thereby providing higher-quality clinical evidence.

**Methods:**

Databases including PubMed, Embase, Cochrane Library, CNKI, and Wanfang were searched for relevant studies published before May 2022. Relevant data were extracted for analysis to compare methodological variables and clinical outcomes.

**Results:**

This meta-analysis included 27 studies with a total of 1,897 patients. In terms of puncture approaches, FO had a significant advantage in reducing VAS at 12 months postoperatively (*P* = 0.019) and efficacy (*P* = 0.043). However, FO performed poorly on complications (*P* < 0.001), operation time (*P* < 0.001), and the number of needle adjustments (*P* < 0.001). Regarding the guidance techniques, the adjunctive use of guidance techniques could reduce patients' 6-month VAS (*P* < 0.001) and 12-month VAS (*P* < 0.001), improve the efficacy (*P* = 0.032), reduce recurrence rates (*P* = 0.001), shorten operation times (*P* < 0.001), decrease times of intraoperative fluoroscopy (*P* < 0.001), and improve the success of the first puncture (*P* < 0.001).

**Conclusion:**

FO radiofrequency thermocoagulation has advantages in efficacy it can still better relieve the pain of patients 12 months postoperatively. However, FO has disadvantages in complications, recurrences, and operation time. The adjunctive use of guidance techniques has a positive effect on treatment efficacy and safety during FO radiofrequency thermocoagulation. However, the results still require large samples and high-quality randomized clinical trials to confirm.

## Introduction

Trigeminal neuralgia (TN) is a common form of paroxysmal craniofacial nerve pain (1). Trigeminal neuralgia occurs in middle-aged and older people, and the incidence increases with age. Moreover, the proportion of female patients is significantly higher than that of male patients (2, 3). TN is an extremely painful disease. Currently, the International Classification of Headache Disorders has classified TN into primary trigeminal neuralgia (PTN) and secondary trigeminal neuralgia (4). PTN patients often present with sudden onset of acute or temporary severe facial pain that severely affects their sleep, diet, and social communication (5, 6).

Treatment of TN is multimodal and includes pharmacotherapy, nerve blocks, radiofrequency thermocoagulation (RFT) of the Gasserian ganglion, and microvascular decompression (MVD) (7, 8). Some patients who become increasingly resistant to pharmacotherapy over time are suitable for more invasive interventions such as microvascular decompression or percutaneous trigeminal nerve ablation (6, 9, 10). RFT is a typical minimally invasive procedure for treating TN due to its advantages of minor trauma and quick recovery (11). Studies have shown that the pain relief rate of RFT is 90% to 100% (12). Percutaneous radiofrequency thermocoagulation of the Gasserian ganglion *via* FO is the most widely used method. However, FO is relatively small and varies in morphology, complicating the cannulation of an already tiny target (13, 14). Anatomical variation of the FO is associated with intraoperative cannulation failure in RFT (15, 16). Therefore, approaches *via* the pterygopalatine fossa (PPF) and foramen rotundum (FR) have been proposed (17, 18). In addition, advances and applications of technologies including computed tomography (CT) guidance, neuronavigation, three-dimensional (3D) navigation techniques, and electrophysiological recordings may enable safer treatment (19–22).

Meta-analysis is a statistically empowering analysis by summarizing different studies to address deficiencies due to small sample sizes and human error (23). It aims to minimize the risk of bias by providing scientific medical evidence. This study evaluated the efficacy and safety of RFT *via* FO for primary trigeminal neuralgia by comparing different approaches and guidance techniques, thereby providing high-quality clinical evidence for TN treatment.

## Method

### Information sources and search strategies

The implementation and reporting of this meta-analysis followed the Preferred Reporting Items for Systematic Reviews (PRISMA) guidelines (24). Two researchers, searched PubMed, Embase, Cochrane Library, CNKI, and China Wanfang. Relevant medical subject headings (MeSH) and keywords were used to identify studies published by July 2022 on the efficacy of foramen ovale RFT in TN. Supplementary Table S1 demonstrates the PubMed search strategy. Search strategies for all databases except PubMed were adapted from the PubMed strategy. Publications from any country/region and written in any language were considered eligible. All identified articles were screened for potentially eligible studies based on title and abstract by a single researcher after removing duplicates and excluding irrelevant studies. Two independent researchers reviewed the full text according to the inclusion and exclusion criteria. Discrepancies were discussed and resolved by consensus with a third researcher if necessary.

### Study selection

**Inclusion criteria:** (1) The study population was adult patients with primary trigeminal neuralgia. All patients underwent percutaneous Gasserian ganglion radiofrequency thermocoagulation. (2) One group of patients underwent radiofrequency thermocoagulation *via* FO. The other group of patients was treated with radiofrequency thermocoagulation *via* a different approach or a different guidance technique. We had no restrictions on approaches or guidance techniques other than FO. (3) Relevant data were reported or provided by the study. Primary data: pain assessment, efficacy, complications, or recurrences. Secondary data: operation time, number of needle adjustments, times of intraoperative fluoroscopy, and the success of the first puncture. (4) The study design was a randomized controlled study or an observational study.

**Exclusion criteria:** (1) The studies were just case reports, systematic reviews, reviews, letters, comments, or conference reports. (2) The Study object was corpses or animals. (3) The studies did not provide sufficient data to calculate effect sizes.

### Data extraction

Two independent researchers extracted study characteristics, demographic variables, methodological variables, and clinical outcome indicators from the studies. Study characteristics extracted included first author, year of publication, study region, sample size, and study type. Demographic variables including gender, age, and disease duration were collected. Methodological variables extracted included approach, guidance techniques, operation time, number of puncture needle adjustments, times of intraoperative fluoroscopy, and the success of the first puncture. Clinical outcome indicators included pain assessment, efficacy, complications, and recurrences. A visual analog scale (VAS) questionnaire was used for pain assessment. Studies that did not use VAS for pain assessment were not involved in the pooled analysis of VAS.

### Quality assessment of included studies

The included studies were assessed by two investigators. The modified Jadad scale was used to assess the quality of randomized controlled trials (RCTs), including the random sequence generation (0–2), randomization (score 0–2), study blinding (score 0–2), and exclusion (score 0–1) (25, 26). Scores 1–3 were considered low quality, and scores 4–7 were considered high quality. Observational studies were assessed using the Newcastle-Ottawa Scale (NOS) (27). A study could only get up to 1 point for each item in the selection and exposure categories and 2 points for each item in the comparability category. Studies with scores of 6–9 were considered high quality (low risk of bias). Studies with scores of 3–5 were considered moderate quality (unclear risk of bias). Studies with scores of 0–2 were considered low quality (high risk of bias) (28).

### Statistical analysis

Statistical analysis was performed using STATA 16.0 software. Relative risks (RR) and corresponding 95% confidence intervals (95% CI) were used to assess the outcome of dichotomous events. For continuous outcomes, we used the weighted mean difference (WMD) and 95% CI for assessment. Heterogeneity was assessed using *I*^2^ statistics (29). *I*^2^ < 40% indicated insignificant heterogeneity. When 40% ≤ *I*^2^ < 60%, moderate heterogeneity was indicated. 60% ≤ *I*^2^ < 90% represented a substantial level of heterogeneity. When 90% ≤ *I*^2^ < 100%, it suggested a considerable level of heterogeneity (30, 31). A random effects model was used when *I*^2^ ≥ 40% and *P* < 0.10. Otherwise, a fixed effects model was used (32). Subgroup analyses were stratified according to different approaches or guidance techniques. Furthermore, when there was significant heterogeneity in the results, we would conduct subgroup analysis based on the number of patients, the ratio of male to female, and the duration of symptoms to explore the source of heterogeneity. Egger's test assessed publication bias when the number of studies involved was ≥ 10. *P* values were calculated using a two-tailed test, and *P* < 0.05 was considered statistically significant.

## Results

### Study selection

**Figure 1:**
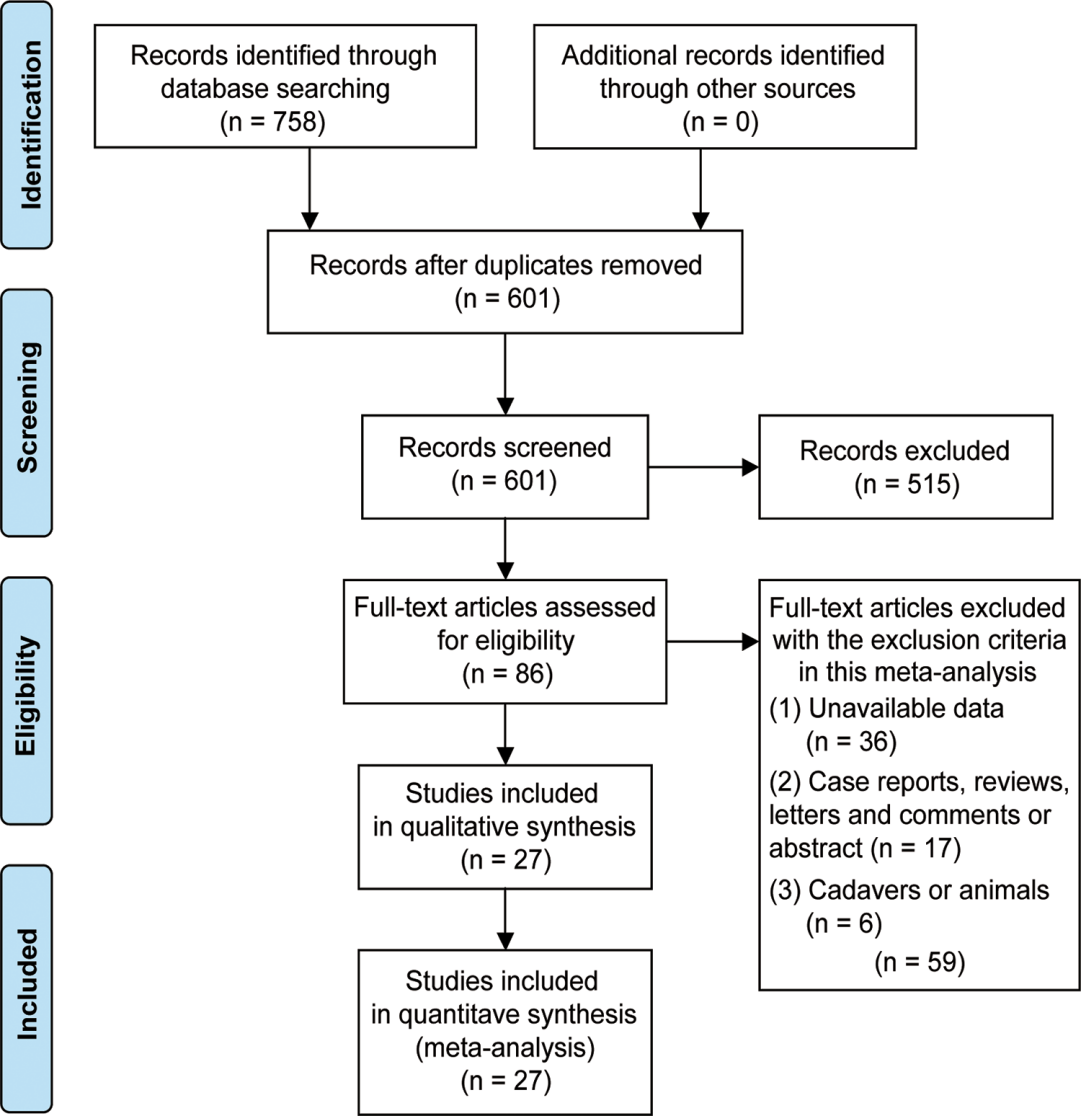
Flow diagram to show the process of the search strategy and relevant results for eligible studies.

Figure 1 illustrated the process of literature search and screening. The search terms were used to identify 758 studies initially. After removing duplicated publications, a total of 518 articles were excluded based on their titles and abstracts. After a full-text review of 86 articles, 27 studies were finally included in the meta-analysis (6, 15, 18, 33–56).

### Quality and characteristics of included studies

The 27 included studies consisted of four randomized controlled trials (6, 38, 44, 56) and 23 observational studies (15, 18, 33–37, 39–43, 45–55). A total of 1,897 patients were included. We obtained gender information from 23 studies with 1,619 patients, including 906 females and 713 males. The mean age of the patients was 61.89 years. The mean duration of the patient's disease was 4.07 years. Patients in all studies were from China. 17 studies compared puncture approaches (15, 18, 35, 38, 40, 42–46, 49–55), and 10 studies compared guidance techniques (6, 33, 34, 36, 37, 39, 41, 47, 48, 56). The mean Jadad score of the four RCTs was 5.75. All observational studies had a NOS score of 6 or higher, and there were three studies with a score of 8, with a mean score of 6.78. Table 1 showed the basic features of the included studies.

**Table 1:** Characteristics and quality assessment of the 22 included studies.

Author	Year	Region	Study design	Patients	Gender (F/M)	Age (years)	Preoperational pain duration (months)	Guidance	Subgroup	JADAD/NOS score
Nie et al.	2014	China	Randomized Controlled Trial	60	36/24	62	26	C-arm x-ray; skin electrical stimulation potentials	Guidance method	5
Huang et al.	2014	China	Prospective study	27	12/15	65	31.9	CT	Puncture approach	6
Ding et al.	2016	China	Randomized Controlled Trial	108	67/41	57.88	90.48	CT and neuronavigation	Puncture approach	6
Xue et al.	2019	China	Prospective study	80	40/40	64.62		DSA	Puncture approach	7
Ding et al.	2021	China	Randomized Controlled Trial	70	41/29	57.68	11.31	CT	Puncture approach	6
He et al.	2022	China	Retrospective study	236	135/101	63.31	64.38	C-arm x-ray	Puncture approach	7
Zhao et al.	2015	China	Prospective study	60	34/26	62.5	26	CT	Puncture approach	7
Fan et al.	2015	China	Prospective study	60	25/35	54.85	72	CT	Puncture approach	8
zhang et al.	2017	China	Prospective study	50	27/23	68.5	17.2	C-arm x-ray; skin electrical stimulation potentials	Guidance method	7
Li et al.	2015	China	Prospective study	100	53/47	55.87	53.64	C-arm x-ray; skin electrical stimulation potentials	Guidance method	7
Liao et al.	2011	China	Prospective study	68	43/25	45	43.2	DSA	Puncture approach	6
Liao et al.	2012	China	Prospective study	42	16/26	73.35	47.14	DSA	Puncture approach	6
Xie et al.	2011	China	Prospective study	30	20/10	66.94	12–72	DSA	Puncture approach	6
Jiang et al.	2012	China	Prospective study	50	31/19	63	5–216	DSA	Puncture approach	7
Cao et al.	2013	China	Prospective study	90	58/32	53.5	NR	DSA	Puncture approach	6
Xiong et al.	2015	China	Prospective study	42	26/16	60.54	NR	C-arm x-ray	Puncture approach	6
Huang et al.	2015	China	Retrospective study	40	24/16	68.1	60	CT	Puncture approach	7
Dong et al.	2018	China	Retrospective study	86	NR	NR	NR	DSA	Puncture approach	7
Chen et al.	2017	China	Prospective study	69	45/24	68.86	NR	x-ray	Puncture approach	8
Yang et al.	2017	China	Prospective study	80	NR	NR	NR	C-arm	Puncture approach	7
Liang et al.	2022	China	Prospective study	68	NR	NR	NR	Ultrasound-guided combined C-arm	Guidance method	7
Zheng et al.	2018	China	Retrospective study	44	NR	NR	NR	Neuronavigation	Guidance method	8
Lu et al.	2015	China	Prospective study	60	36/24	62.15	5.25	CT; 3D printing navigation template	Guidance method	6
Zheng et al.	2022	China	Prospective study	60	35/25	65	5.5	x-ray; 3D printing navigation template	Guidance method	7
Xu et al.	2006	China	Randomized Controlled Trial	54	27/27	60.93	5.09	neuronavigator-guided	Guidance method	6
Zhao et al.-1	2021	China	Retrospective study	110	66/44	58.23	NR	3D printing navigation template	Guidance method	6
Zhao et al.-2	2021	China	Prospective study	53	21/32	65.61	NR	3D printing navigation template	Guidance method	7

F, female; M, male; CT, computed tomography; NR: not reported.

### Meta-analysis for comparison of puncture approaches

**Figure 2:**
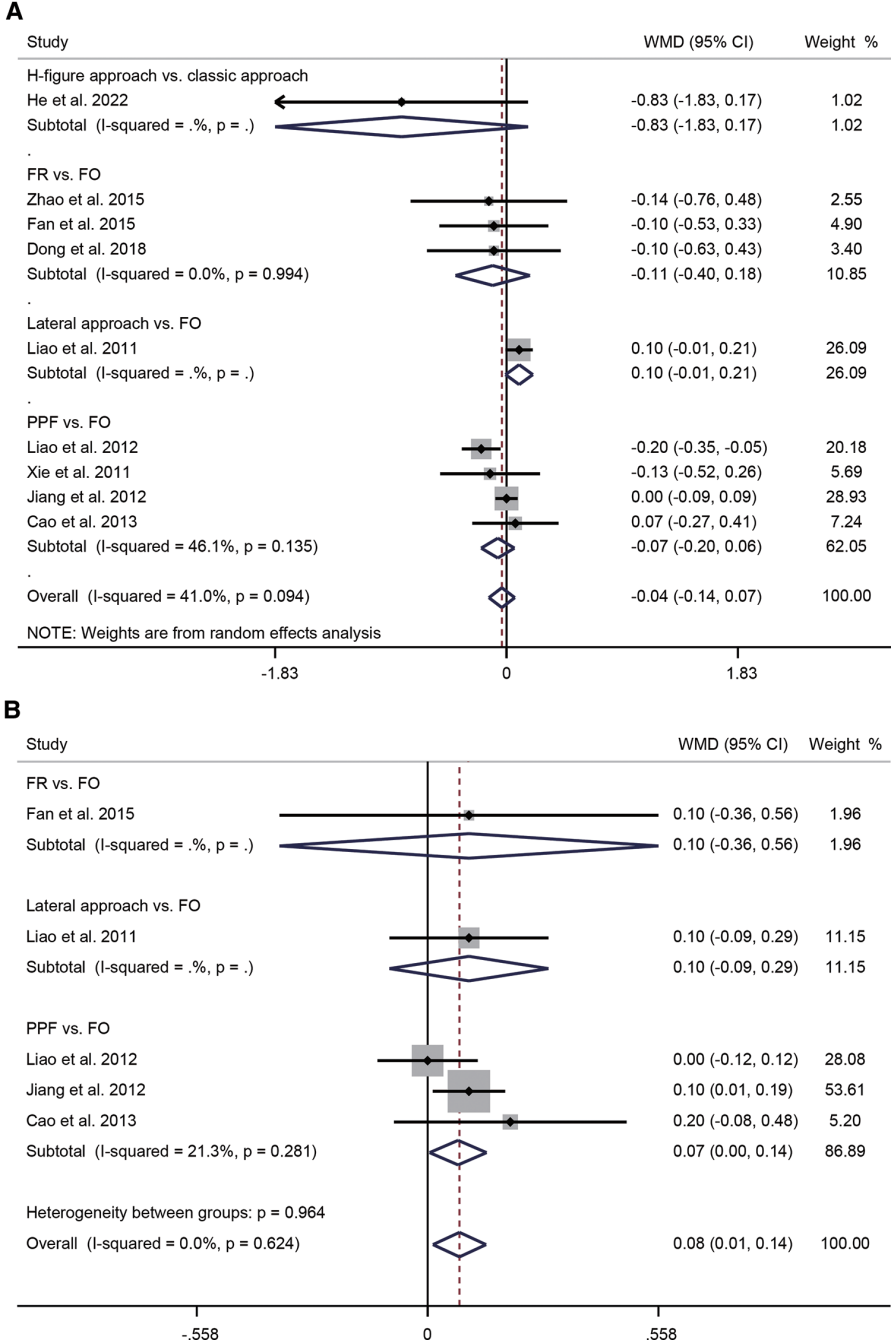
Forest plot of VAS at 6 months (**A**) and VAS at 12 months postoperatively (**B**) for the comparison of different puncture approaches (other puncture approaches vs. FO). VAS, visual analog scale; FO, foramen ovale.

Seventeen studies compared puncture approaches, including the FR, mandibular angle, H-figure fluoroscopic landmark, and PPF. Nine studies reported patients' VAS at six months postoperatively. Due to moderate heterogeneity (*I*^2^ = 41.0%, *P* = 0.094), a random-effects model was applied. Meta-analysis suggested no statistically significant difference between FO and other puncture approaches (WMD = −0.04, 95% CI: −0.14–0.07, *P* = 0.480). Subgroup analysis showed a WMD of −0.83 for H-figure fluoroscopic landmark, −0.11 for FR, 0.10 for lateral approach, and −0.07 for PPF (Figure 2A). To explore the source of heterogeneity, we performed subgroup analysis based on the number of patients, the ratio of male to female and the duration of symptoms. The results suggested that the number of patients and the ratio of male to female may be the source of heterogeneity (Supplementary Table S2). Five studies reported patients' VAS at 12 months postoperatively. Studies showed the lowest level of heterogeneity (*I*^2^ = 0.0%, *P* = 0.624), so a fixed effect model was used. The pooled WMD was 0.08 (95% CI: 0.01–0.14, *P* = 0.019), indicating that VAS was significantly lower in FO than in other approaches at 12 months. Subgroup analysis showed a WMD of 0.10 for FR, 0.10 for lateral approach, and 0.07 for PPF (Figure 2B).

**Figure 3:**
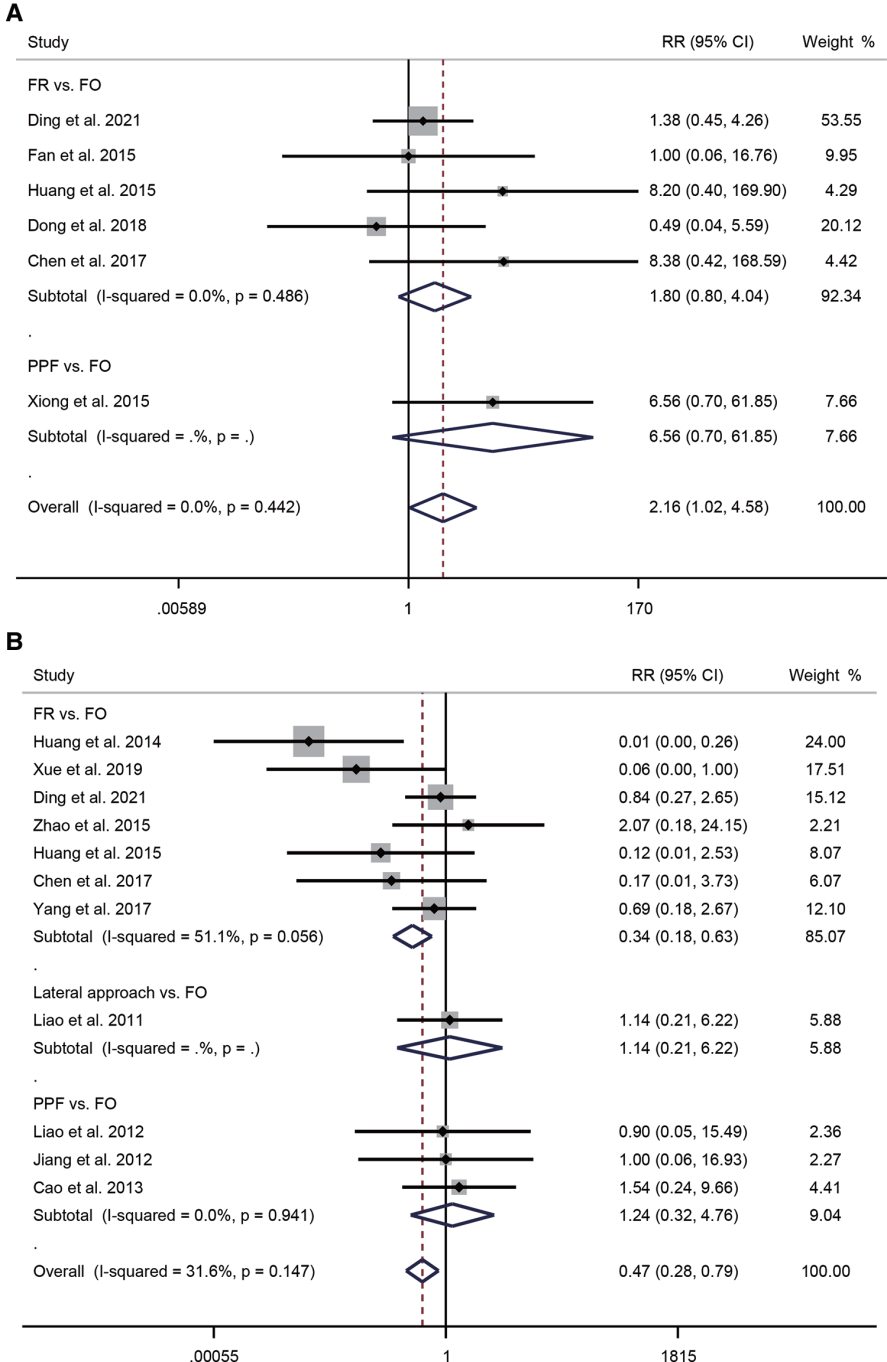
Forest plot of efficacy (**A**) and recurrence rate (**B**) for the comparison of different puncture approaches (other puncture approaches vs. FO). FO, foramen ovale.

**Figure 4:**
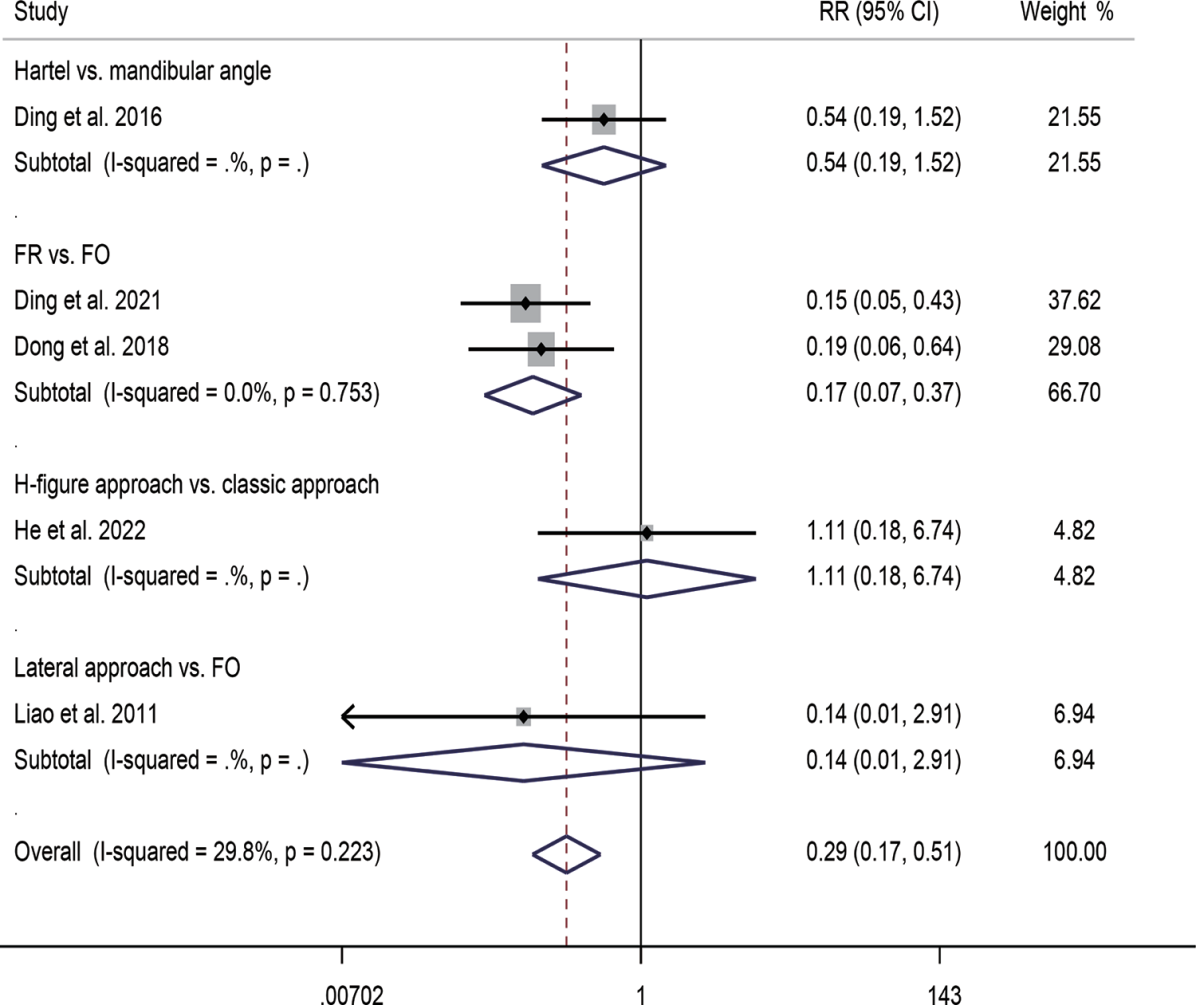
Forest plot of complication for the comparison of different puncture approaches (other puncture approaches vs. FO). FO, foramen ovale.

In terms of efficacy, a total of six studies were reported. The pooled results of the fixed-effects model (*I*^2^ = 0.0%, *P* = 0.442) indicated that patients in the FO approach had higher efficacy (RR = 2.16, 95% CI: 1.02–4.58, *P* = 0.043). Subgroup analysis showed an RR of 1.8 for FR and 6.56 for PPF (Figure 3A). Eleven studies were included in calculating the recurrence rate. The results showed insignificant heterogeneity between studies (*I*^2^ = 31.6%, *P* = 0.147). The pooled results from the fixed-effects model demonstrated that patients receiving FO had a higher recurrence rate compared with other approaches (RR = 0.47, 95% CI: 0.28–0.79, *P* = 0.004). Subgroup analysis showed an RR of 0.34 for FR, 1.14 for lateral approach, and 1.24 for PPF (Figure 3B). Five studies provided data on complications. The pooled results showed insignificant heterogeneity (*I*^2^ = 29.8%, *P* = 0.223). The combined RR was 0.29 (95% CI: 0.17–0.51, *P* < 0.001), indicating that patients undergoing FO had a higher complication rate. Subgroup analysis showed an RR of 0.54 for mandibular angle, 0.17 for FR, 1.11 for H-figure fluoroscopic landmark, and 0.14 for lateral approach (Figure 4).

**Figure 5:**
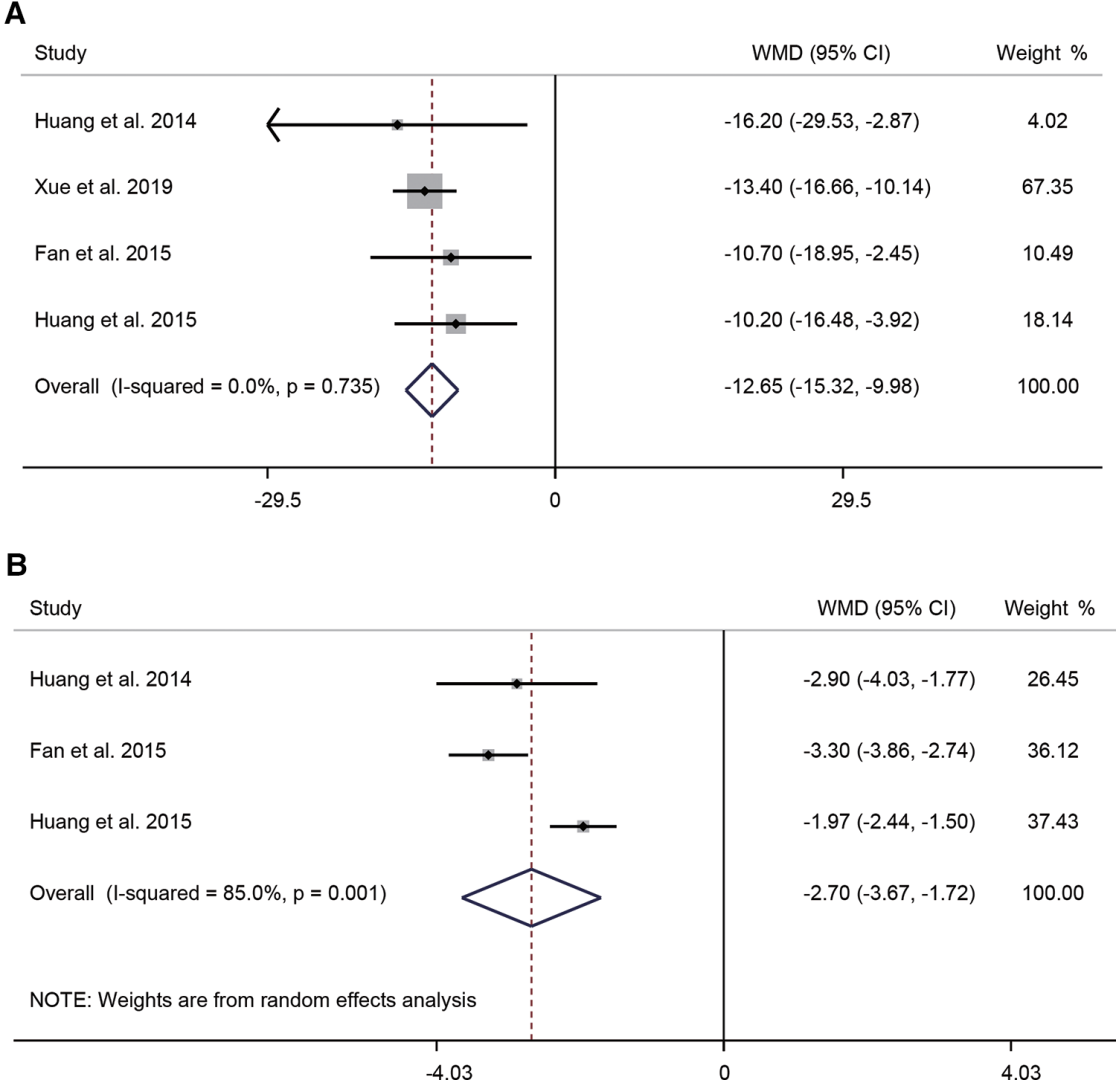
Forest plot of operation time (**A**) and number of needle adjustments (**B**) for the comparison of different puncture approaches (other puncture approaches vs. FO). FO, foramen ovale.

Four studies compared the operation times of the FO and FR approaches. A fixed-effects model (*I*^2^ = 0.0%, *P* = 0.735) indicated that FO required longer operation time compared with FR (WMD = −12.65, 95% CI: −15.32–−9.98, *P* < 0.001) (Figure 5A). Three studies reported the number of needle adjustments. The substantial level of heterogeneity in the results (*I*^2^ = 85.0%, *P* = 0.001) was analyzed using a random effects model. The pooled WMD was −2.70 (95% CI: −3.67–−1.72, *P* < 0.001), indicating that the number of needle adjustments was significantly higher in patients receiving FO than in those receiving FR (Figure 5B). The results of subgroup analyses according to the number of patients and the ratio of male to female showed that the ratio of male to female may be a source of heterogeneity (Supplementary Table S3).

### Meta-analysis of guidance techniques

**Figure 6:**
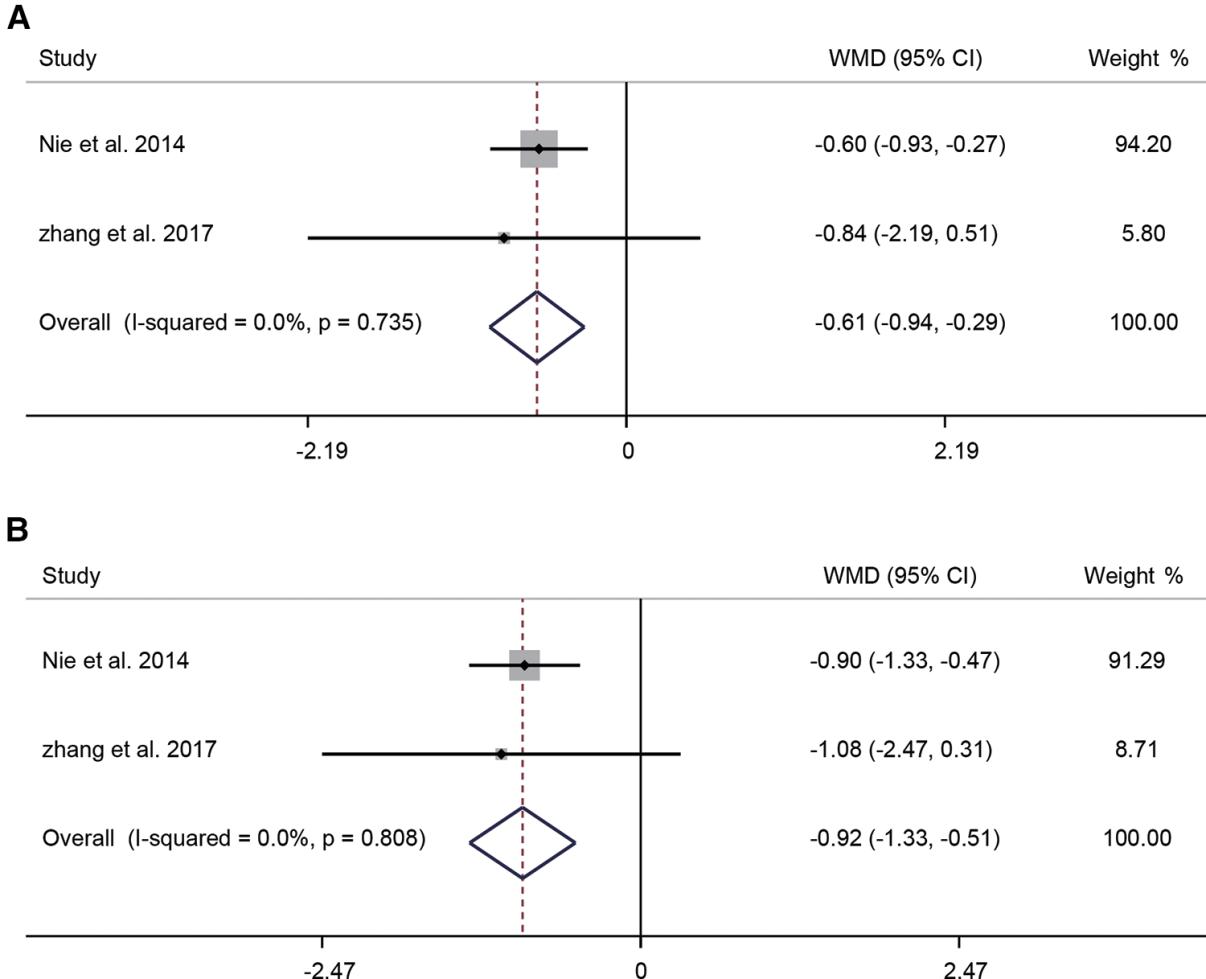
Forest plot of VAS at 6 months (**A**) and VAS at 12 months postoperatively (**B**) for the comparison of different guidance techniques (combined with other guidance techniques vs. simple image guidance). VAS, visual analog scale.

Two of the ten studies that compared guidance techniques reported VAS in patients at 6 and 12 months postoperatively. Due to minimal heterogeneity (*I*^2^ = 0.0%, *P* = 0.735), a fixed-effects model was used. The pooled results showed that at 6 months postoperatively, the use of x-ray imaging combined with skin stimulation potential guidance significantly reduced patients' VAS scores (WMD = −0.61, 95% CI: −0.94–−0.29, *P* < 0.001) (Figure 6A). The aggregated results of the fixed-effects model (*I*^2^ = 0.0%, *P* = 0.808) showed that the use of x-ray imaging combined with skin stimulation potential guidance significantly reduced patients' VAS scores at 12 months postoperatively (WMD = −0.92, 95% CI: −1.33–−0.51, *P* < 0.001) (Figure 6B).

**Figure 7:**
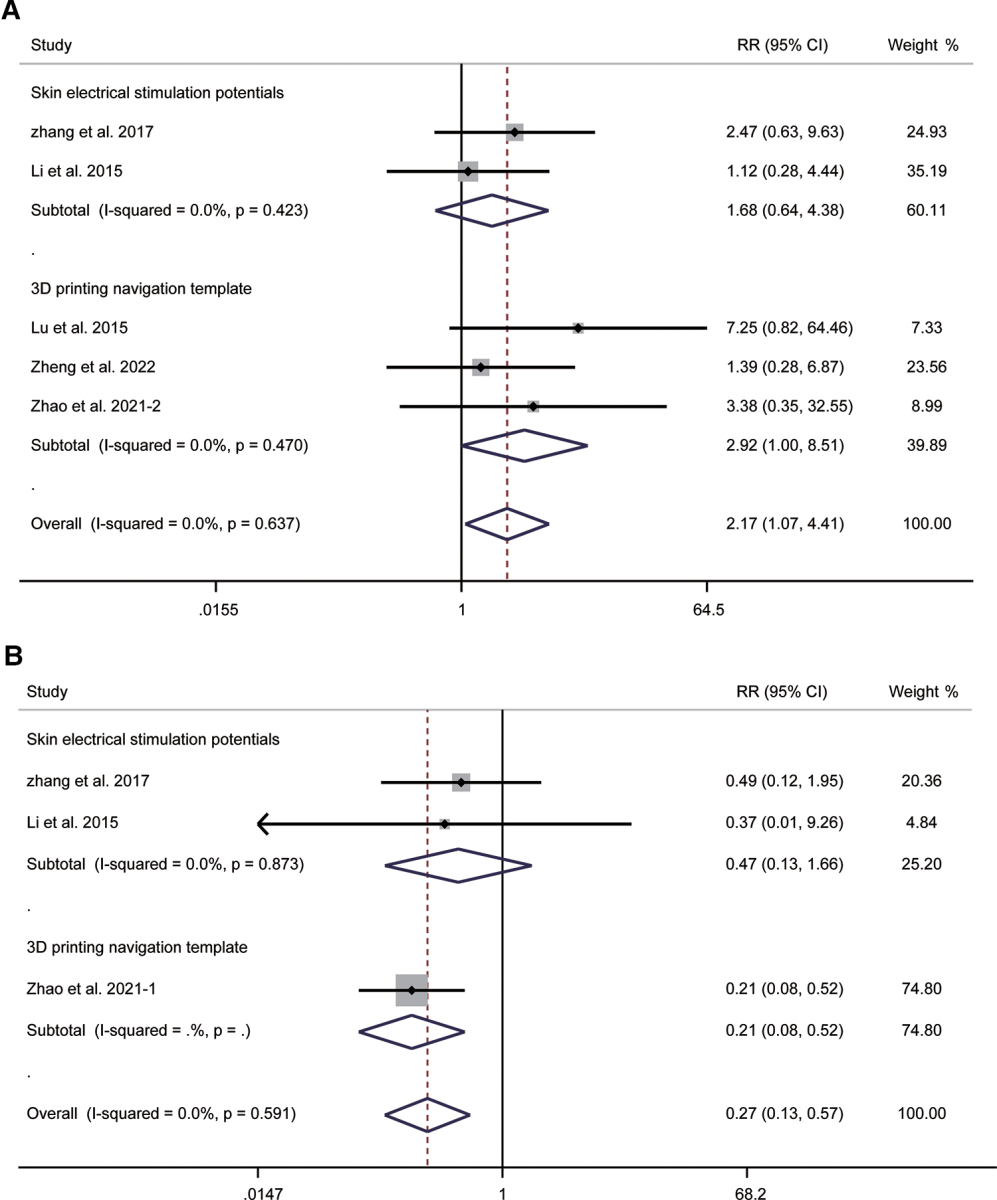
Forest plot of efficacy (**A**) and recurrence rate (**B**) for the comparison of different guidance techniques (combined with other guidance techniques vs. simple image guidance).

Five studies involving 323 patients provided data regarding efficacy. The pooled results without significant heterogeneity (*I*^2^ = 0.0%, *P* = 0.637) suggested that the assisted guidance techniques could improve the efficacy of FO radiofrequency thermocoagulation (RR = 2.17, 95% CI: 1.07–4.41, *P* = 0.032). Subgroup analysis showed an RR of 1.68 (95% CI: 0.64–4.38, *P* = 0.289) for combined skin electrical stimulation potentials and 2.92 (95% CI: 1.00–8.51, *P* = 0.050) for 3D printing navigation template (Figure 7A). Three studies were included in calculating the recurrence rate. The summary results demonstrated no significant heterogeneity (*I*^2^ = 0.0%, *P* = 0.591). The pooled RR was 0.27 (95% CI: 0.13–0.57, *P* = 0.001), indicating that the combination of guidance techniques could significantly reduce the recurrence rate. Subgroup analysis showed an RR of 0.47 (95% CI: 0.13–1.66, *P* = 0.238) for combined skin electrical stimulation potentials and 0.21 (95% CI: 0.08–0.52, *P* = 0.001) for 3D printing navigation template (Figure 7B).

**Figure 8:**
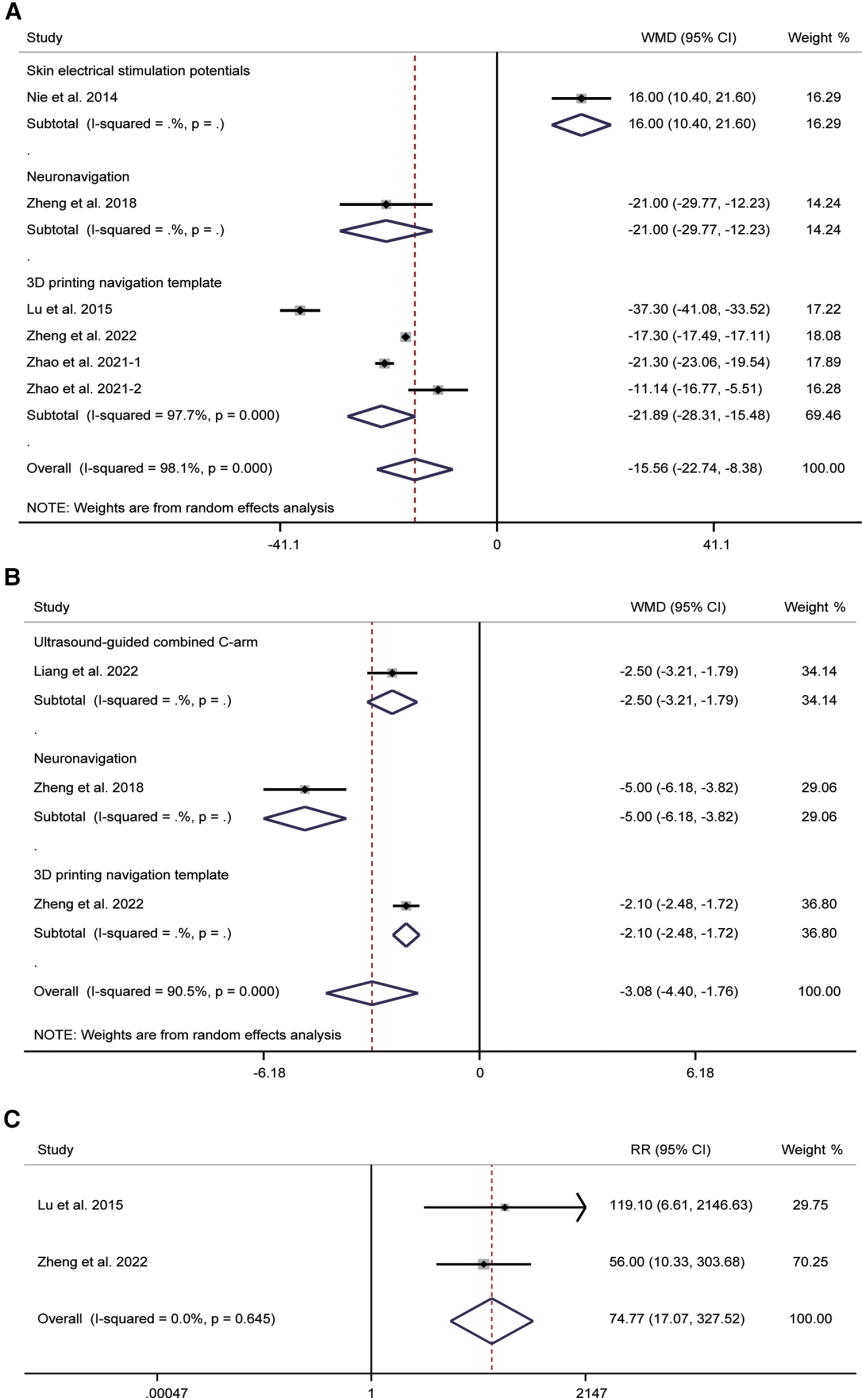
Forest plot of operation time (**A**), times of intraoperative fluoroscopy (**B**), and the success of the first puncture (**C**) for the comparison of different guidance techniques (combined with other guidance techniques vs. simple image guidance).

A total of six studies compared operation times. Pooled results of the random-effects model (*I*^2^ = 97.9%, *P* < 0.001) indicated that operation times could be shortened by combining guidance techniques (WMD = −15.56, 95% CI: −22.74–−8.38, *P* < 0.001). Subgroup analysis showed a WMD of 16.00 (95% CI: 10.40–21.60, *P* < 0.001) for combined skin electrical stimulation potentials, −21.00 (95% CI: −29.77–−12.23, *P* < 0.001) for neuronavigation, and −21.89 (95% CI: −28.31–−15.48, *P* < 0.001) for 3D printing navigation template (Figure 8A). In addition, due to significant heterogeneity, we performed subgroup analysis based on the number of patients, the ratio of male to female and the duration of symptoms. The results showed that all of them may not be the source of heterogeneity (Supplementary Table S4).

For times of intraoperative fluoroscopy, three studies involving 172 patients were reported. The most significant level of heterogeneity in the pooled results (*I*^2^ = 90.5%, *P* < 0.001) was analyzed using a random-effects model. The pooled WMD was −3.08 (95% CI: −4.40–−1.76, *P* < 0.001), indicating a reduction in times of intraoperative fluoroscopy with the use of guidance techniques. Subgroup analysis showed a WMD of −2.50 (95% CI: −3.21–−1.79, *P* < 0.001) for ultrasound-guided combined C-arm, −5.00 (95% CI: −6.18–−3.82, *P* < 0.001) for neuronavigation, and −2.10 (95% CI: −2.48–−1.72, *P* < 0.001) for 3D printing navigation template (Figure 8B). The results of subgroup analysis based on the number of patients suggested that the number of patients may be a source of heterogeneity (Supplementary Table S5).

Two studies provided data related to the success of the first puncture. Pooled results showed no significant heterogeneity between studies (*I*^2^ = 0.0%, *P* = 0.645). The pooled RR was 74.77 (95% CI: 17.07–327.52, *P* < 0.001), indicating that 3D printing navigation template assisted with radiofrequency thermocoagulation of FO may improve the accuracy of FO puncture (Figure 8C).

### Publication bias

The results of Egger's test demonstrated no significant publication bias in recurrence for puncture approaches (*P* = 0.094).

## Discussion

TN is a common facial pain syndrome. RFT *via* FO is the most commonly used treatment for TN, providing successful pain relief in approximately 80%–98% of patients (57, 58). This systematic review and meta-analysis aimed to assess the efficacy and safety of radiofrequency thermocoagulation *via* FO for trigeminal neuralgia by both the puncture approach and the guidance techniques. Concerning the puncture approach, our results suggested that radiofrequency thermocoagulation *via* FO had greater efficacy. Compared to 6 months postoperatively, FO radiofrequency thermocoagulation could provide better pain relief to patients at 12 months postoperatively. However, FO could lead to higher complications and recurrence rates, and longer operation times. In terms of guidance techniques, the use of x-ray imaging combined with skin electrical stimulation potentials during FO radiofrequency thermocoagulation reduced the VAS scores of patients at 6 and 12 months postoperatively. The assisted guidance techniques during FO radiofrequency thermocoagulation resulted in better clinical outcomes, including reducing recurrence rates, shortening operation times, decreasing times of intraoperative fluoroscopy, and improving the success of the first puncture.

The FO is an oval opening in the posterior part of the pterygoid bone, which is posterior and medial to the carotid canal (59). The FO's shape varies, leading to technical difficulties in cannulating the FO. In the Gasserian ganglion, the three nerve fiber branches of the trigeminal nerve are in close contact and partially interconnected. Anatomically, because of the difficulty in locating the maxillary division (V2), the ophthalmic division (V1) and mandibular division (V3) are quickly involved when the RF needle is passed through the FO, resulting in complications including masticatory weakness and facial numbness (18, 38, 60). Several studies have shown fewer complications with RFT *via* FR than FO (15, 38, 45, 50). Xie et al., Cao et al., and Xiong et al. found that PPF has a lower complication rate than FO (46, 51, 54). It may be because the FR or PPF approach is shifting the target of radiofrequency thermocoagulation from the semilunar ganglion to the V2 cranial branch, from intracranial to extracranial procedures, significantly reducing the damage to the middle meningeal artery, optic nerve and other trigeminal branches during radiofrequency puncture (61). Some studies have shown a lower incidence of postoperative headaches in patients using FR (18, 49, 50). It may be because FR avoids repeated punctures and needle adjustments. Liao et al. concluded that the lateral FO approach is feasible and has fewer surgical complications than FO (55). In addition, some studies have proposed a mandibular angle approach (44). The trajectory is almost parallel to the coronal plane using the mandibular angle approach, avoiding contact with the infratemporal fossa. Thus, it reduces the possibility of injury to the maxillary artery. Ding et al. showed that the conventional Hartel and submandibular approaches could complement each other in overcoming the technical difficulties associated with the anatomic variation of the FO (44).

In RFT, the thermal coagulation point is determined based on the anatomical location of the RF needle tip and the patient's response to electrical stimulation to avoid the unintentional neurolytic block of unaffected branches (58). However, in clinical practice, patient discomfort and incorrect responses often lead to errors and biases in physician judgment, leading to increased complications or even treatment failure (62, 63). Our results showed that a combination of guidance techniques such as stimulation potential guidance, ultrasound, neuronavigation, and 3D printing navigation template might improve the effectiveness of treatment and reduce repeated operations. A prospective study showed that using x-ray imaging for initial localization and then determining the exact location of each branch of the trigeminal nerve based on electrical stimulation can improve the accuracy of RF thermocoagulation site localization, improve short- and medium-term clinical outcomes, and reduce short-term surgical complications (6). Zheng et al. concluded that FO radiofrequency thermocoagulation for TN can encourage neuronavigation assistance with better operational efficiency and less radiation exposure (39). With the growing maturity of computer-aided design and manufacturing of 3D digital human technology, the application of 3D printing navigation template in FO puncture has developed rapidly in recent years. The skull base and skin were reconstructed according to the preoperative cranial CT images to precisely locate the FO and mark it as the puncture target. The best puncture approach and depth are designed and a digital navigation model is formed. The individualized navigation templates are obtained through 3D printing. Therefore, compared with traditional FO radiofrequency thermocoagulation, 3D printing navigation template can improve the accuracy of FO puncture and reduce the occurrence of postoperative complications due to the deviation of puncture direction and inaccurate depth (33, 36, 37, 47). The use of assisted guiding techniques makes it possible to eliminate the dependence on the surgeon's skill and experience in the puncture.

## Strengths and limitations of the study

This meta-analysis has several strengths. First, the number of included studies was relatively large. Therefore, we could assess the differences between FO and other puncture approaches with relatively high accuracy in different subgroups of puncture approaches. In addition, most of the included studies were prospective studies. Thus, the results were less likely to be influenced by recall or selection bias. Furthermore, we used a sensitive search strategy with no language restrictions to minimize publication bias and to identify as many relevant studies as possible.

In contrast, this study also has some limitations. First, the review was potentially biased due to the limitations of the researcher in the literature search and independent data extraction and analysis. Moreover, the quality of the included literature was relatively low. Only three of the studies in this study were randomized controlled trials; the rest were cohort studies. Most cohort studies were of low quality with small and unequal sample sizes, potentially leading to confounding bias. The included studies have differences in the number of patients, the ratio of male to female, and the duration of symptoms, which may be the source of heterogeneity. Although the random-effects model was used, heterogeneity still existed. The meta-analysis only included results that had appeared in two or more studies. Consequently, many other high-quality studies were not included in the meta-analysis due to incomplete data. This study did not compare the advantages of different puncture approaches and guidance techniques. Furthermore, the small sample size was a significant limitation of this meta-analysis. Only three of all included studies had a sample size of more than 100 cases. Therefore, the conclusions would be limited. In addition, the study area covered only China and did not include studies from other countries, which might affect the adaptability of the conclusions to other populations. Lastly, the differences in surgical methods and functional assessment criteria between studies might lead to bias.

## Clinical and research significance

This systematic review and meta-analysis summarized the efficacy of radiofrequency thermocoagulation *via* FO for TN. Although the complications and recurrences of FR, PPF, mandibular angle, and lateral approaches were lower than FO, their efficacy and pain relief were also reduced. When combined with additional guidance techniques, Gasserian ganglion radiofrequency coagulation had an encouraging effect. The critical goal of clinical practice is to reduce the difficulty of surgery, obtain better treatment effects and improve patient satisfaction. This study provided more reliable evidence that clinicians can draw upon when developing surgical plans. Future studies could explore in depth the impact of specific puncture approaches and guidance techniques on the clinical outcomes of Gasserian ganglion radiofrequency thermocoagulation to provide accurate puncture localization and cannulation approach. Therefore, studies with large samples and well-designed randomized controlled trials are required. In addition, the current research evaluated the efficacy and safety of FO radiofrequency thermocoagulation on TN according to the puncture approaches and guidance techniques, but the advantages of the different puncture approaches and guided techniques were not known to us. In a follow-up study, we can explore the advantages of different puncture approaches and guided techniques through a network meta-analysis.

## Conclusion

radiofrequency thermocoagulation *via* FO is an effective strategy for treating trigeminal neuralgia. Compared with other puncture approaches, FO has advantages in efficacy, and it can still better relieve the pain of patients 12 months postoperatively. However, FO radiofrequency thermocoagulation increases the complications and recurrence rates in patients. The adjunctive use of guidance techniques during FO radiofrequency thermocoagulation can lead to better efficacy, reduce patient pain, reduce recurrence rates, shorten operation times, decrease times of intraoperative fluoroscopy, and improve the success of the first puncture. However, a large, well-designed, high-quality randomized clinical trial with a large sample is still needed to explore the specific puncture approach and the effect of the guidance techniques on FO radiofrequency thermocoagulation.

## Data Availability

The original contributions presented in the study are included in the article/**Supplementary Material**, further inquiries can be directed to the corresponding author/s.
