# Supplementary material for: Puncture approaches and guidance techniques of radiofrequency thermocoagulation through foramen Ovale for primary trigeminal neuralgia: Systematic review and meta-analysis
Source: Front Surg. 2023 Jan 6;9:1024619. doi: 10.3389/fsurg.2022.1024619 (PMC9853901; doi:10.3389/fsurg.2022.1024619)
Supplement: Supplementary file 1 [file Table1.docx]

Supplementary Table 1. PubMed search strategy.

| # | Searches |
| --- | --- |
| 1 | “radiofrequency thermocoagulation”[Title/Abstract] OR “frequency radiation thermocoagulation”[Title/Abstract] OR “radio-frequency”[Title/Abstract] OR “radiofrequency ablation”[Title/Abstract] OR “continuous radiofrequency”[Title/Abstract] |
| 2 | “Trigeminal Neuralgia”[Mesh] |
| 3 | “trigeminal neuralgia”[Title/Abstract] OR “trigeminal neuropathic pain”[Title/Abstract] OR “orofacial pain”[Title/Abstract] OR “fothergill disease”[Title/Abstract] OR “gasserian ganglion”[Title/Abstract] OR “trifacial neuralgia”[Title/Abstract] OR “trifacial neuralgias”[Title/Abstract] OR “idiopathic trigeminal neuralgia”[Title/Abstract] OR “primary trigeminal neuralgia”[Title/Abstract] |
| 4 | #2 OR #3 |
| 5 | “Foramen Ovale”[Mesh] |
| 6 | “foramen ovale”[Title/Abstract] OR “cranial channel”[Title/Abstract] OR “oval foramen”[Title/Abstract] OR “FO”[Title/Abstract] |
| 7 | #5 OR #6 |
| 8 | #1 AND #4 AND #7 |
