# Supplementary material for: Puncture approaches and guidance techniques of radiofrequency thermocoagulation through foramen Ovale for primary trigeminal neuralgia: Systematic review and meta-analysis
Source: Front Surg. 2023 Jan 6;9:1024619. doi: 10.3389/fsurg.2022.1024619 (PMC9853901; doi:10.3389/fsurg.2022.1024619)
Supplement: Supplementary file 2 [file Table2.doc]

**Supplementary Table 2. Subgroup analysis of** **VAS at 6 months according to the number of patients, duration, and the ratio of male to female.**

| Subgroup | WMD (95% CI) | Heterogeneity I^2^ (%), *P* |
| --- | --- | --- |
| **The number of patients:** | | |
| ≥ 100 | -0.83 (-1.83, 0.17) | NA |
| 50-100 | 0.07 (-0.02, 0.17) | 0.0%, *P* = 0.786 |
| ≤ 50 | -0.09 (-0.25, 0.17) | 60.8%, *P* = 0.078 |
| **Duration:** | | |
| > 5 | -0.32 (-0.98, 0.34) | 42.0%, *P* = 0.189 |
| ≤ 5 | -0.06 (-0.31, 0.20) | 80.1%, *P* = 0.007 |
| Unclear | -0.00 (-0.09, 0.08) | 0.0%, *P* = 0.872 |
| **The ratio of male to female:** | | |
| F > M | 0.03 (-0.06, 0.11) | 15.0%, *P* = 0.318 |
| F < M | -0.19 (-0.33, -0.04) | 0.0%, *P* = 0.668 |
| Unclear | -0.10 (-0.63, 0.43) | NA |

WMD, weighted mean difference; CI, confidence interval; F, female; M, male.
