# Supplementary material for: Puncture approaches and guidance techniques of radiofrequency thermocoagulation through foramen Ovale for primary trigeminal neuralgia: Systematic review and meta-analysis
Source: Front Surg. 2023 Jan 6;9:1024619. doi: 10.3389/fsurg.2022.1024619 (PMC9853901; doi:10.3389/fsurg.2022.1024619)
Supplement: Supplementary file 3 [file Table3.doc]

**Supplementary Table 3. Subgroup analysis of needle adjustments according to the number of patients and the ratio of male to female.**

| Subgroup | WMD (95% CI) | Heterogeneity I^2^ (%), *P* |
| --- | --- | --- |
| **The number of patients:** | | |
| ≤ 50 | -2.29 (-3.15, -1.42) | 55.3%, *P* = 0.135 |
| 50-100 | -3.30 (-3.86, -2.74) | NA |
| **The ratio of male to female:** | | |
| F < M | -3.22 (-3.72, -2.72) | 0.0%, *P* = 0.533 |
| F > M | -197 (-2.44, -1.50) | NA |

WMD, weighted mean difference; CI, confidence interval; F, female; M, male.
