# Supplementary material for: Puncture approaches and guidance techniques of radiofrequency thermocoagulation through foramen Ovale for primary trigeminal neuralgia: Systematic review and meta-analysis
Source: Front Surg. 2023 Jan 6;9:1024619. doi: 10.3389/fsurg.2022.1024619 (PMC9853901; doi:10.3389/fsurg.2022.1024619)
Supplement: Supplementary file 4 [file Table4.doc]

**Supplementary Table 4. Subgroup analysis of** **operation times according to the number of patients, duration, and the ratio of male to female.**

| Subgroup | WMD (95% CI) | Heterogeneity I^2^ (%), *P* |
| --- | --- | --- |
| **The number of patients:** | | |
| 50-100 | -12.59 (-27.92, 2.75) | 98.8%, *P* < 0.001 |
| ≤ 50 | -21.00 (-29.77, -12.23) | NA |
| ≥ 100 | -21.30 (-23.06, -19.54) | NA |
| **Duration:** | | |
| > 5 | -0.32 (-0.98, 0.34) | 99.1%, *P* < 0.001 |
| ≤ 5 | -0.06 (-0.31, 0.20) | NA |
| Unclear | -0.00 (-0.09, 0.08) | 82.5%, *P* = 0.003 |
| **The ratio of male to female:** | | |
| F > M | -15.44 (-24.42, -6.46) | 98.8%, *P* < 0.001 |
| F < M | -11.14 (-16.77, -5.51) | NA |
| NA | -21.00 (-29.77, -12.23) | NA |

WMD, weighted mean difference; CI, confidence interval; F, female; M, male.
