# Supplementary material for: Puncture approaches and guidance techniques of radiofrequency thermocoagulation through foramen Ovale for primary trigeminal neuralgia: Systematic review and meta-analysis
Source: Front Surg. 2023 Jan 6;9:1024619. doi: 10.3389/fsurg.2022.1024619 (PMC9853901; doi:10.3389/fsurg.2022.1024619)
Supplement: Supplementary file 5 [file Table5.doc]

**Supplementary Table 5. Subgroup analysis of** **times of intraoperative fluoroscopy according to the number of patients.**

| Subgroup | WMD (95% CI) | Heterogeneity I^2^ (%), *P* |
| --- | --- | --- |
| **The number of patients:** | | |
| 50-100 | -2.19 (-2.52, -1.85) | 0.0%, *P* = 0.332 |
| ≤ 50 | -5.00 (-6.18, -3.82) | NA |

WMD, weighted mean difference; CI, confidence interval; F, female; M, male.
